# Structural Connectivity in Gilles de la Tourette Syndrome

**DOI:** 10.15844/pedneurbriefs-29-4-1

**Published:** 2015-04

**Authors:** Ana B. Chelse, Joanna S. Blackburn

**Affiliations:** 1 Division of Neurology, Ann & Robert H. Lurie Children's Hospital of Chicago, Chicago, IL; 2 Departments of Pediatrics and Neurology, Northwestern University Feinberg School of Medicine, Chicago, IL

**Keywords:** Gilles de la Tourette Syndrome, Cortico-Basal Ganglia Networks, Structural Connectivity, Tractography

## Abstract

Investigators from Centre de Reference National Maladie Rare ‘Syndrome Gilles de la Tourette’ and Sorbonne University report white matter abnormalities in the pathways connecting the cerebral cortex, basal ganglia, and thalamus in a group of 49 adults with Tourette syndrome (TS).

Investigators from Centre de Reference National Maladie Rare ‘Syndrome Gilles de la Tourette’ and Sorbonne University report white matter abnormalities in the pathways connecting the cerebral cortex, basal ganglia, and thalamus in a group of 49 adults with Tourette syndrome (TS). High-resolution diffusion tensor imaging and fractional anisotropy probabilistic techniques were utilized to examine the connectivity of white matter tracts in the cortico-striato-pallido-thalamo-cortico (CSPTC) networks. Patients with TS had white matter abnormalities in neuronal pathways connecting the striatum, basal ganglia, and thalamus to the cerebral cortex, including the primary motor cortex, primary somatosensory cortex, and supplementary motor area. The reported enhanced connectivity of the motor cortex was positively associated with increased motor tic severity, regardless of medication, age, or gender. The cortico-striatal pathways showed elevated fractional anisotropy and diminished radial diffusivity, suggesting microstructural abnormalities in the white matter tracts. Authors reported that these abnormal connections were predominantly observed in females when compared to males. [1]

COMMENTARY. The mechanism of TS is complex and not fully understood. Neuro-pathological, electrophysiological, and functional neuroimaging studies have led to increased knowledge in understanding the dysfunctional neuronal processing of CSPTC networks in TS. Recent advances in diffusion tensor imaging (DTI) enhanced our ability to investigate white matter structures and tracts in the basal ganglia. Prior research using DTI showed abnormalities in the white matter tracts of the somatosensory, fronto-striatal and motor pathways in TS [2, 3]. Alterations in the microstructure of white matter tracts have also been described in adults and adolescents with TS, specifically reduced white matter connectivity in the corpus callosum. This reduction in white matter microstructure was associated with reductions in tic severity [4–6]. The current study adds important information to this body of literature. The authors showed enhanced connectivity in the CSPTC white matter pathways of the motor cortex that was positively associated with increased motor tic severity. Additionally, authors reported that these abnormal connections were predominantly in females. These data may suggest that women with TS are less likely to have resolution of their tics in adulthood when compared to men. Interestingly, Lichter et al. reported that women with TS tend to have more disruption in function from their tics when compared to men [7]. It may be that the increased connectivity in the CSPTC pathways in women with Tourette syndrome contributes to their reported increased tic burden. In light of new technology, future DTI studies could further elucidate the pathophysiology and abnormal trajectory of white matter tracts in adults and adolescents with TS and how it relates to tic severity.

## References

[1] Worbe Y, Marrakchi-Kacem L, Lecomte S, Valabregue R, Poupon F, Guevara P (2015). Altered structural connectivity of cortico-striato-pallido-thalamic networks in Gilles de la Tourette syndrome. Brain.

[2] Govindan RM, Makki MI, Wilson BJ, Behen ME, Chugani HT (2010). Abnormal water diffusivity in corticostriatal projections in children with Tourette syndrome. Hum Brain Mapp.

[3] Neuner I, Kupriyanova Y, Stocker T, Huang R, Posnansky O, Schneider F (2010). White-matter abnormalities in Tourette syndrome extend beyond motor pathways. Neuroimage.

[4] Plessen KJ, Bansal R, Peterson BS (2009). Imaging evidence for anatomical disturbances and neuroplastic compensation in persons with Tourette syndrome. J Psychosom Res.

[5] Draper A, Stephenson MC, Jackson GM, Pepes S, Morgan PS, Morris PG (2014). Increased GABA contributes to enhanced control over motor excitability in Tourette syndrome. Curr Biol.

[6] Jackson SR, Parkinson A, Jung J, Ryan SE, Morgan PS, Hollis C (2011). Compensatory neural reorganization in Tourette syndrome. Curr Biol.

[7] Lichter DG, Finnegan SG (2015). Influence of gender on Tourette syndrome beyond adolescence. Eur Psychiatry.

